# Temperature-induced structural changes in thermosensitive transient receptor potential channels (thermoTRPs)

**DOI:** 10.3389/fnmol.2026.1911902

**Published:** 2026-08-17

**Authors:** Lina Ni

**Affiliations:** School of Neuroscience, Virginia Polytechnic and State University, Blacksburg, VA, United States

**Keywords:** cryo-EM, thermoreceptor, thermoTRP, transient receptor potential channel, TRPM8, TRPV1, TRPV3

## Abstract

The ability to sense environmental temperature is fundamental to animal survival, physiological homeostasis, and adaptation to changing environments. Animals rely on temperature-responsive molecules to detect changes in environmental and internal temperatures to maintain their thermal homeostasis. Among these molecules, the best-characterized group belongs to the transient receptor potential (TRP) channel superfamily, commonly referred to as thermoTRPs. ThermoTRPs have been extensively studied and are well established as thermoreceptors. In recent years, cryo-electron microscopy (cryo-EM) has enabled the structural characterization of numerous thermoTRP channels in their closed-state and agonist-induced open state, providing unprecedented insights into their architecture and gating mechanisms. Despite these advances, the molecular mechanisms by which thermoTRPs undergo temperature-induced activation remain poorly understood. This review focuses on six pioneering cryo-EM studies reporting temperature-induced open structures to summarize current structural evidence and discuss potential mechanisms by which temperature drives channel opening in thermoTRPs.

## Introduction

Temperature is a fundamental environmental variable that animals continuously encounter and that profoundly impacts their physiology and pathophysiology (Franks and Hoffmann, 2012; Smith, 2013; Baldwin et al., 2023; Vay et al., 2012). To maintain thermal homeostasis and sustain normal physiological functions, animals rely on temperature-responsive molecules that detect environmental and internal temperature changes (Mota-Rojas et al., 2021; Morrison and Nakamura, 2019). Among the temperature-responsive molecules identified to date, the largest and best-characterized group belongs to the transient receptor potential (TRP) channel superfamily, commonly referred to as thermoTRPs. These receptors exhibit remarkable sensitivity to temperature, with each responding to a distinct temperature range that together cover the physiologically relevant range of temperatures from noxious cold ( < 15 °C) to noxious heat ( > 43 °C) (Clapham, 2003; Dhaka et al., 2006; Patapoutian et al., 2003; Bandell et al., 2007; Ramsey et al., 2006; Kashio and Tominaga, 2022; Vriens et al., 2014; Julius, 2013). For example, heat-sensitive vanilloid channels (TRPV1-4) detect warmth and noxious heat (Caterina et al., 1997, 1999, 2000; Peier et al., 2002b; Xu et al., 2002; Smith et al., 2002; Güler et al., 2002), while melastatin channels (TRPM) include both cold-sensitive member TRPM8 (McKemy et al., 2002; Peier et al., 2002a) and heat-sensitive channels (Talavera et al., 2005; Vriens et al., 2011; Tan and McNaughton, 2016; Song et al., 2016; Vandewauw et al., 2018). Although thermoTRPs have been extensively studied and well-identified as thermoreceptors, the molecular mechanisms by which thermoTRPs undergo temperature-dependent activation remain incompletely understood.

To address this question, substantial efforts have been devoted to identifying the structural determinants of temperature sensing in thermoTRPs through mutagenesis studies (Zubcevic et al., 2019; Liu and Qin, 2017; Vlachová et al., 2003; Yao et al., 2011; Grandl et al., 2010, 2008; Brauchi et al., 2006; Zhang F. et al., 2018; Gregorio-Teruel et al., 2015; Kim et al., 2013; Macikova et al., 2019; Laursen et al., 2016; Liu and Qin, 2021; Cordero-Morales et al., 2011; Ladrón-de-Guevara et al., 2020; Kim et al., 2020; Yang et al., 2010; Susankova et al., 2007; Myers et al., 2008). The results generally indicate that temperature sensitivity depends on contributions from multiple domains distributed across the entire channel, including the ankyrin repeat domains, the coupling domain, the S1-S4 domain, the pore domain, and the C-terminal domain, supporting the hypothesis that temperature sensing arises from global conformational changes across the entire protein (Feng, 2014). However, they do not exclude the possibility that localized temperature sensors initiate and subsequently propagate these global structural rearrangements.

In fact, mutagenesis studies also demonstrate that individual amino acid substitutions can alter thermosensitivity. In mouse TRPA1, three residues (S250, M258, and D261) within ankyrin repeat six are necessary for cold sensitivity. Substitution of each individual residue (S250N, M258L, or D261G) renders mouse TRPA1 responsive to warm temperatures, suggesting that these residues contribute to the thermal tuning of TRPA1 (Jabba et al., 2014). Similarly, avian TRPM8 is temperature insensitive, whereas human TRPM8 exhibits robust cold sensitivity. A single point mutation in the pore loop (Y905V) confers strong cold sensitivity to the avian channel, while the reciprocal mutation reduces temperature sensitivity of human TRPM8 (Choi et al., 2026). Several critical residues have also been identified in TRPV1. For example, the N331K mutation in ankyrin repeat five broadly affects human TRPV1 sensitivity to temperature, pH, and chemical stimuli, indicating that this residue contributes to general channel gating (Katz et al., 2023). Other residues play more specific roles in thermosensation. In mouse TRPV1, mutations at S124N in ankyrin repeat one and E188Q in the linker between ankyrin repeats two and three significantly increase the activation threshold (Laursen et al., 2016), whereas mutations R151Q and K154L in ankyrin repeat one decrease the activation threshold of rat TRPV1 (Hori et al., 2023). These findings suggest that specific residues and their interaction networks can tune thermoTRP activation. However, mutagenesis studies alone cannot determine whether these residues function as primary temperature sensors or as critical nodes that propagate temperature-induced conformational rearrangements.

The development of cryo-electron microscopy (cryo-EM) has greatly advanced our understanding of TRP channels. Cryo-EM has emerged as a powerful structural biology technique for determining high-resolution structures of membrane proteins (Kühlbrandt, 2014; Cheng, 2018). Since 2013, when the first structures of the TRPV1 channel were determined by cryo-EM (Liao et al., 2013; Cao et al., 2013), numerous structures of diverse TRP channels have been reported, including multiple thermoTRP channels (Huynh et al., 2016; Zubcevic et al., 2016, 2019, 2018; Shimada et al., 2020; Deng et al., 2020; Singh et al., 2018; Yin et al., 2018; Guo et al., 2017; Winkler et al., 2017; Autzen et al., 2018; Zhang Z. et al., 2018; Huang et al., 2018; Wang et al., 2018; Tang et al., 2018; Fan et al., 2018; Azumaya et al., 2018; Paulsen et al., 2015; Hirschi et al., 2017; Chen et al., 2017; Schmiege et al., 2017; Shen et al., 2016; Deng et al., 2018; Yin et al., 2025; Ruan et al., 2021). ThermoTRPs are polymodal ion channels that integrate diverse physical and chemical stimuli, including temperature, pH, osmolarity, membrane voltage, and natural and synthetic chemical agonists (Fernández-Ballester et al., 2023). For example, besides high temperature, TRPV1 is activated by capsaicin, acidic pH, and membrane depolarization (Caterina et al., 1997; Tominaga et al., 1998; Voets et al., 2004). In addition to closed-state structures, agonist-induced open-state structures of thermoTRPs have also been resolved, providing important insights into agonist-dependent gating mechanisms (Cao et al., 2013; Singh et al., 2018; Kwon et al., 2023; Huang et al., 2018; Yin et al., 2025; Karuppan et al., 2024; Yin et al., 2019; Suo et al., 2020). Despite these advances, structural characterization of temperature-induced open states remains poorly understood.

To elucidate the molecular mechanisms by which thermoTRPs detect temperature changes and undergo temperature-dependent activation, it is essential to characterize their temperature-induced open states. In this review, we focused on thermoTRPs whose temperature-induced open structures were captured by cryo-EM. A comprehensive literature search was conducted on April 13, 2026, using PubMed with search terms targeting (1) TRP channels or thermoreceptors, (2) cryo-EM structural studies, and (3) temperature-dependent activation or thermosensation. Available preprints were also considered. Studies were included if cryo-EM structures captured temperature-induced open structure of thermoTRP channels. Structures obtained in the presence of agonists, sensitizing mutations, or species variants were included when the open structure was induced by temperature. Studies were excluded if they predated the first TRP channel cryo-EM structures (Liao et al., 2013; Cao et al., 2013), were review articles, did not include cryo-EM structural studies, did not investigate temperature-dependent channel activation, or did not capture temperature-induced open structures. Based on these criteria, six cryo-EM studies capturing temperature-induced open structures of thermoTRP channels were identified (Supplementary Table 1), including two wild-type TRP channels (TRPV3 and TRPM8) (Choi et al., 2026; Nadezhdin et al., 2021), two sensitized TRPV3 mutants (Singh et al., 2019; Mugo et al., 2025), and two agonist-bound open states (TRPV1 bound to capsaicin and TRPM8 bound to menthol) (Lee et al., 2025; Kwon et al., 2021). This review focuses on these six pioneering studies to summarize current structural evidence and discuss potential mechanisms by which temperature induces channel opening.

## Structural domains of TRPV1, TRPV3, and TRPM8

**FIGURE 1:**
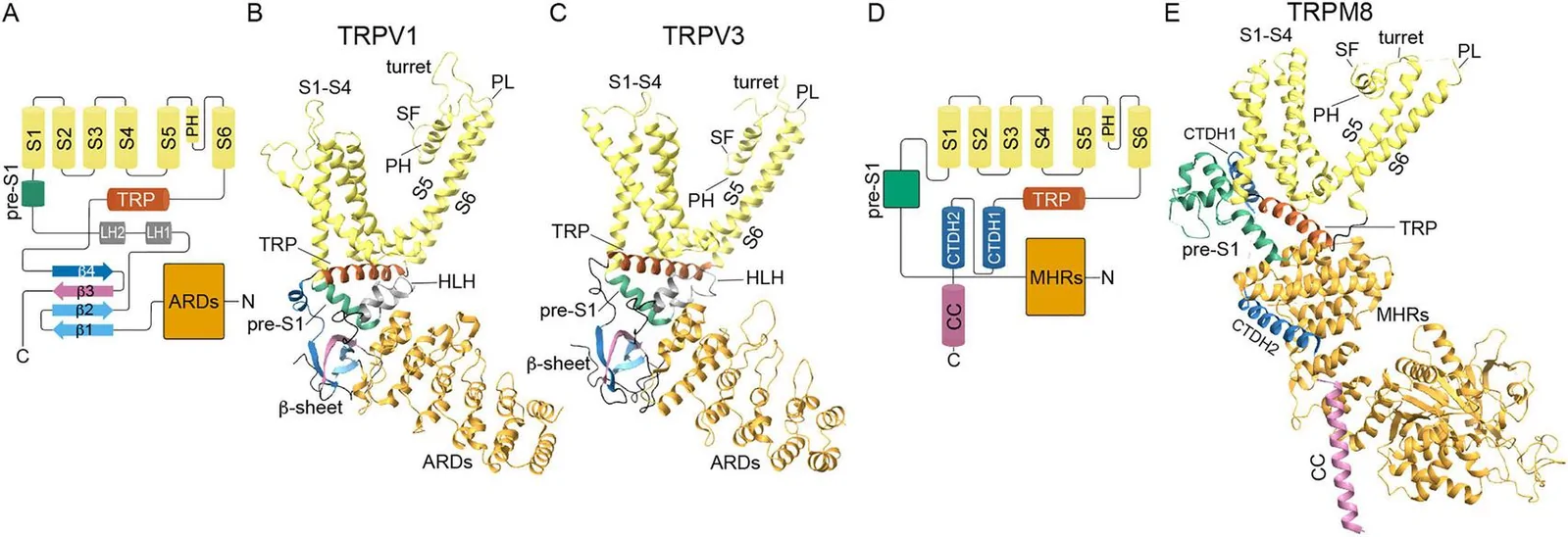
Architecture of a single subunit of TRPV1, TRPV3, and TRPM8. **(A,D)** Schematic domain organization of a single subunit of TRPV **(A)** and TRPM8 **(D).** **(B,C,E)** Structures of a single subunit of TRPV1 [**(B)**, PDB 7LP9], TRPV3 [**(C)**, PDB 7MIJ], and TRPM8 [**(E)**, PDB 9P8Y). From the N-terminus to the C-terminus, TRPV1 and TRPV3 comprise six ankyrin repeat domains (ARDs), a β-turn (β1 and β2), a helix-loop-helix motif (HLH; LH1 and LH2), a pre-S1 helix (pre-S1), six transmembrane helices (S1–S6), a TRP helix (TRP), and a β-turn (β3 and β4). The ARDs, N-terminal β-turn, HLH, and pre-S1 form the N-terminal domain (NTD). S1–S6 constitute the transmembrane domain (TMD). S5, S6, and the structural elements between them, including the turret, pore helix (PH), selectivity filter (SF), and pore loop (PL), constitute the pore domain (PD). The TRP helix and C-terminal β-turn form the C-terminal domain (CTD). From the N-terminus to the C-terminus, TRPM8 comprises four melastatin homology regions (MHRs), a pre-S1 domain (pre-S1), six transmembrane helices (S1–S6), a TRP helix (TRP), two C-terminal helices (CTDH1 and CTDH2), and a coiled-coil domain (CC). The MHRs and pre-S1 form the NTD. S1-S6 constitute the TMD. S5, S6, and the structural elements between them, including the turret, PH, SF, and PL, constitute the PD. The TRP helix, CTDH1, CTDH2, and CC form the CTD.

TRPV1 and TRPV3 share a similar overall architecture. Both channels are homotetramers, with each subunit consisting of an N-terminal domain (NTD), a transmembrane domain (TMD) containing six transmembrane helices (S1-S6), and a C-terminal domain (CTD) (Figure 1A–C). The NTD includes the cytosolic ankyrin repeat domains (ARDs) and a coupling domain (CD). The ARDs and TMD are connected by the CD, which consists of a β-turn, a helix-loop-helix motif (HLH), and a pre-S1 helix. The TMD comprises the S1-S4 bundle and a pore domain (PD). The PD involves S5, the turret, pore helix (PH), selectivity filter (SF), pore loop (PL), and S6. The CTD includes an amphipathic TRP helix and a β-turn that forms a β-sheet with the N-terminal β-turn (Kwon et al., 2021; Liao et al., 2013; Zubcevic et al., 2018; Cao et al., 2013).

TRPM8 is also a homotetrameric channel, with each subunit comprising an NTD, a TMD, and a CTD (Figures 1D, E). The NTD includes the cytosolic melastatin homology region (MHRs; MHR1-MHR4) and pre-S1 domain. The architecture of the TMD in TRPM8 is similar to that of TRPV1 and TRPV3. In the CTD, the TRP helix is followed by two C-terminal helices (CTDH1 and CTDH2) and a coiled-coil domain (CC) (Lee et al., 2025; Yin et al., 2018).

Within the PD, the SF and S6 form the upper and lower constrictions, respectively. In TRPV1 and TRPV3, both the SF and S6 contribute to channel gating by forming two distinct constriction sites (Nadezhdin et al., 2021; Singh et al., 2019; Kwon et al., 2021). In TRPM8, however, gating is primarily mediated by S6, which forms a single constriction site at the lower gate (Choi et al., 2026; Lee et al., 2025).

## Heat-induced gating of TRPV3

The Sobolevsky group has published two studies that provide important insights into the mechanisms underlying heat-induced opening of mouse TRPV3 (Singh et al., 2019; Nadezhdin et al., 2021). Although the open structure reported by Singh et al. was obtained using a sensitized mutant channel (Y564A) (Singh et al., 2019), Nadezhdin et al. subsequently captured the open-state structure of wild-type TRPV3 (Nadezhdin et al., 2021). In this review, we discuss these two studies together to provide an overview of the structural basis of heat-induced TRPV3 activation.

In the closed state, S6 adopts an α-helical or a π-helical conformation, depending on the membrane environment. Two lipid-binding sites are present within the TMD. At Site 1, a lipid is wedged between the extracellular portions of S4 and S6. At Site 2, the lipid inserts its headgroup into a crevice formed by the S2-S3 linker, S4, the S4-S5 linker, and the TRP helix. The CD contains an 11-residue insertion that interacts with the C-terminal region. The C-terminus forms a coil that wraps around the β-sheet and partially occupies a cleft formed at the interface between intracellular domains of adjacent subunits, where it establishes contacts with the ARDs of a neighboring subunit (Singh et al., 2019; Nadezhdin et al., 2021).

Heat-induced activation of TRPV3 occurs through two sequential steps: a strongly temperature-dependent first step followed by a weakly temperature-dependent second step (Singh et al., 2019; Nadezhdin et al., 2021). Heating induces dissociation of lipids from both binding sites, thereby promoting closer association between S4 and S6. This closer association drives tilting of S6 toward S4 and an α-to-π transition with S6, followed by an approximately 100° axial rotation of its C-terminal half and concomitant tilting of the TRP helix. Subsequently, S6 elongates by two helical turns, while the TRP helix shortens by two helical turns (Singh et al., 2019; Nadezhdin et al., 2021).

**FIGURE 2:**
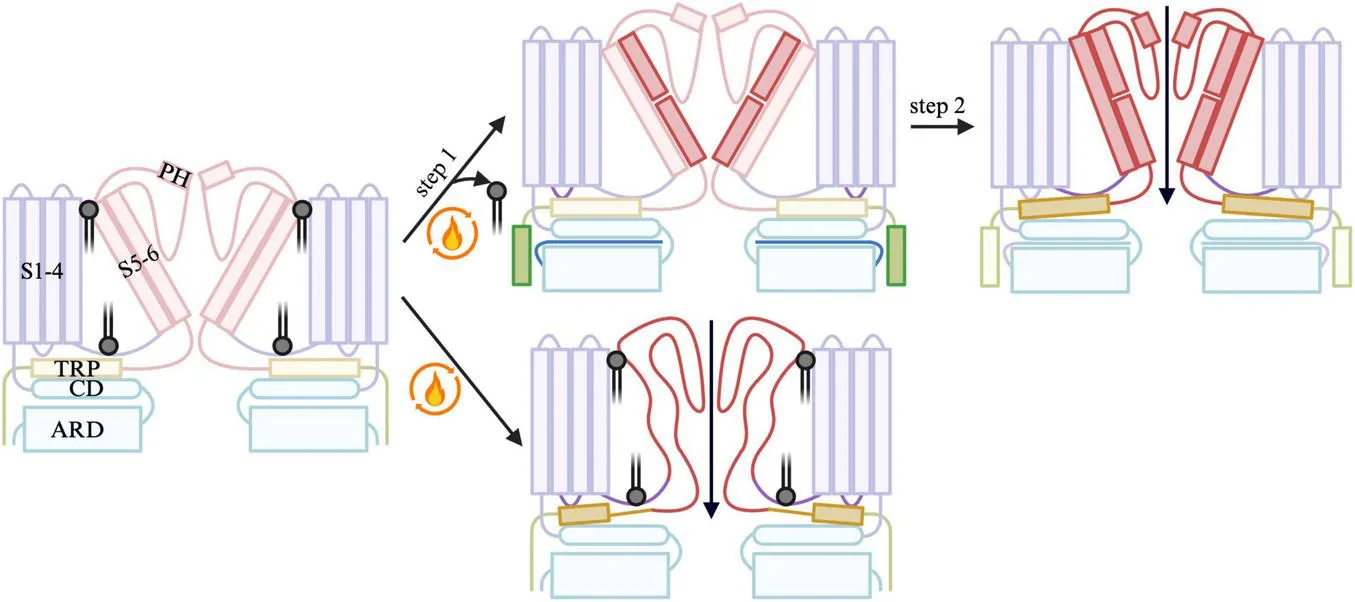
Heat-induced gating of TRPV3. Two mechanisms have been proposed. In the first mechanism (upper panel), heat triggers lipid dissociation, an α-to-π transition in the S6 helices, changes in the α-helicity of the S2-S3 linker, rearrangement of the C-terminal CC domain into an α-helix, folding of the N-terminal polypeptide, and rigid-body movements of the ARDs and the CD (step 1). These conformational changes result in an upward translation of the S4-S5 linker, S5, S6, and the N-terminal portion of the TRP helix, opening the channel (step 2). The second mechanism (lower panel) involves disruption of the interaction network linking the TRP helix, S6, and the S4-S5 linker and the interaction network between the S2-S3 linker and the CD, causing partial unfolding of the S4-S5 linker, S5, S6, and the N-terminal portion of the TRP helix, which removes the channel constriction and enables pore opening. Domains involved in the conformational changes are highlighted in darker colors.

Heating induces both rigid-body movements and secondary-structure rearrangements. While rigid-body movements involve the ARDs, CD, and β-sheet, secondary-structure changes occur in the S2-S3 linker, C terminus, and N terminus. The S2-S3 linker undergoes redistribution of α-helicity between its N- and C-terminal portions. The C terminus unwraps from the β-sheet, transitions from a coil to an α-helix, lines the wall of an intracellular cleft, and establishes new contacts with neighboring subunits. The N terminus becomes ordered and inserts into the same cleft. These rearrangements pull the β-sheet of one subunit toward the ARDs of a neighboring subunit, thereby narrowing the intersubunit cleft and strengthening intersubunit interactions. Collectively, the intracellular domains rotate by approximately 8° and move toward the transmembrane domain, shortening the overall height of the channel by approximately 5 Å (Figure 2; Singh et al., 2019; Nadezhdin et al., 2021).

During the weakly temperature-dependent second step, the 11-residue insertion in the CD disappears. The S4-S5 linker moves upward, followed by S5, S6, and the N-terminal portion of the TRP helix, leading to dilation of the SF and S6 and channel opening (Figure 2; Singh et al., 2019; Nadezhdin et al., 2021).

Besides the two studies described above, Mugo et al. also determined structures of closed wild-type mouse TRPV3, as well as closed, heat-induced intermediate, and heat-induced open states of a sensitized mutant channel (TRPV3-412S, a single-residue insertion in the interhelical loop of HLH in the CD) (Mugo et al., 2025).

Mugo et al. found that the wild-type closed structure features a hydrogen bond between the TRP helix and the adjacent S6-TRP helix linker, a salt bridge between S6 and the S4-S5 linker, and a hydrogen bond between the S4-S5 linker and the TRP helix, stabilizing the closed structure of the PD and the intersubunit interface. In addition, the HLH in the CD interacts with the S2-S3 linker, the ARDs, and the TRP helix, supporting interactions at the interface between the TMD and the intracellular domains. Furthermore, Mugo et al. observed three lipid-like densities. Besides the two lipid-binding sites reported by Singh et al. and Nadezhdin et al., a ring-shaped density is positioned laterally beneath the PH, with finger-like extensions projecting into intersubunit spaces between S6 and the adjacent PD (Singh et al., 2019; Nadezhdin et al., 2021; Mugo et al., 2025).

In the non-heated TRPV3-412S channel, conformational changes are observed at the intersubunit interface and interface between the TMD and the intracellular domains. The hydrogen bond between the TRP helix and the adjacent S6-TRP helix linker is weakened, impacting interactions at the intersubunit interface. This change is accompanied by remodeling of the HLH in CD, in which the C-terminal helix elongates, and the interhelical loop shortens, weakening interactions among the HLH, S2-S3 linker, ARDs, and TRP helix, and leading to rearrangements at the interface between the TMD and the intracellular domains. These changes result in partial unwinding at the S6 junction and slight elongation of the S6-TRP helix linker, which shifts upward toward the gate (Mugo et al., 2025).

Heating induces conformational changes of the PD. At its N-terminal side, the salt bridge between the S4-S5 linker and S6 and the hydrogen bond between the S4-S5 linker and the TRP helix are disrupted, resulting in fusion of the S4-S5 linker and S5 into a continuous helix. At the C-terminal side of the PD, the N-terminal portion of the TRP helix becomes disordered, extending the S6-TRP helix linker. Consistent with the study by Nadezhdin et al., heating induces the S2-S3 linker to adopt a helical conformation, thereby impacting the interface between the TMD and the intracellular domains. Collectively, these structural rearrangements lead to mild vertical compression and lateral contraction of the channel. Interestingly, heating does not induce lipid dissociation in the TRPV3-412S channel (Figure 2; Singh et al., 2019; Nadezhdin et al., 2021; Mugo et al., 2025).

Mugo et al. identified a heat-induced state in which the PD density is absent and interpreted the loss of density as evidence of tertiary structural collapse or partial unfolding of the S4-S5 linker, S5, S6, and the N-terminal portion of the TRP helix (Figure 2). Although Singh et al. also reported a similar state, they attributed the missing density to pronounced conformational heterogeneity rather than structural collapse (Singh et al., 2019; Mugo et al., 2025). The basis for the absence of PD density remains unclear and requires further investigation. Of note, both structures lacking PD density are observed in sensitized mutant channels.

## Heat-induced gating of TRPV1

Kwon et al. used the rat TRPV1 to determine the open-state structure in the presence of capsaicin. Although temperature and chemical agonists can activate TRPV1 independently, they also allosterically modulate one another. For example, subthreshold concentrations of capsaicin lower its temperature activation threshold (Voets et al., 2004). Compared with the closed state, capsaicin binding induces S2, the S2-S3 linker, the intracellular portion of S3, and the S4-S5 linker move toward one another, but does not produce substantial changes in the SF or S6 gates (Kwon et al., 2021).

**FIGURE 3:**
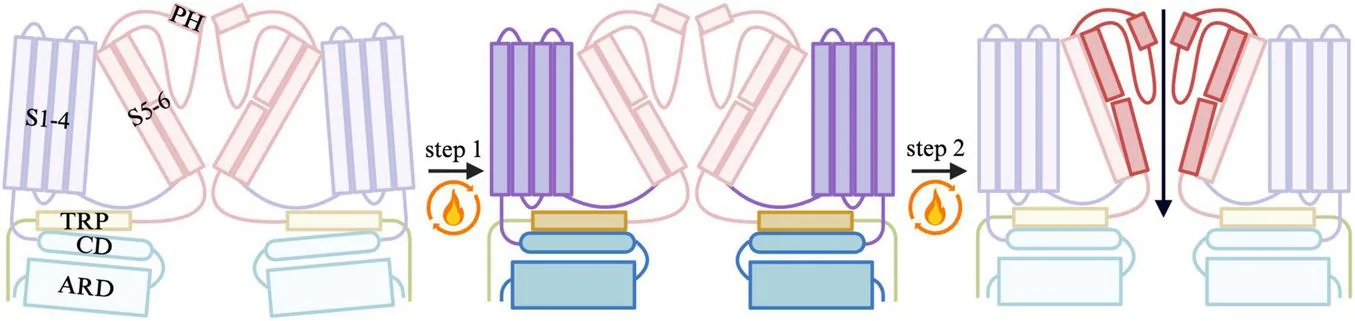
Heat-induced gating of TRPV1. In step 1, global conformational rearrangements occur in the ARD, CD, S1-S4, and TRP domains, leading to channel contraction. In step 2, local conformational changes in the outer pore and S6 helices induce dilation of the SF and S6 gate, resulting in channel opening. Domains involved in the conformational changes are highlighted in darker colors.

Heating induces global conformational changes involving the intracellular domains and the S1-S4 bundle. Within the intracellular region, the distal ARDs (ARD1-ARD3) become unresolved, while the CD and proximal ARDs (ARD4-ARD6) undergo a rigid-body rotation toward the channel. This movement brings the HLH in the CD to closer to the TRP helix. Within the TMD, the S1-S4 bundle and the C-terminal portion of the S4-S5 loop rotate toward the pore, resulting in a slight dilation of the S6 gate (Figure 3; Kwon et al., 2021).

Heating also drives rearrangements of the outer pore (Figure 3). The outer pore of each subunit rotates around the membrane, accompanied by substantial movements of the PL and turret junction and subtle movements of the PH and SF. This arrangement disrupts an interaction network involving the turret junction, PL, and S1, leading to slight dilation of both the SF and S6 gates (Kwon et al., 2021).

Finally, heating induces rearrangement of a π helix-like segment within S6, leading to rotation of the lower portion of S6 (Figure 3). This rotation is stabilized or induced by rotamer changes between S6 and the S4-S5 linker, which are associated with conformational changes of the S4-S5 linker, neighboring S5, and neighboring S6, leading to changes in intersubunit interaction and opening of the lower gate (Kwon et al., 2021).

## Cold-induced gating of TRPM8

Choi et al. recently resolved the structures of avian and human TRPM8 (Choi et al., 2026). Because avian TRPM8 is insensitive to cold temperatures (Yang et al., 2020), this review focuses on human TRPM8. By combining cryo-EM with hydrogen-deuterium exchange mass spectrometry (HDX-MS), the authors proposed a structural mechanism for cold-induced activation of human TRPM8.

**FIGURE 4:**
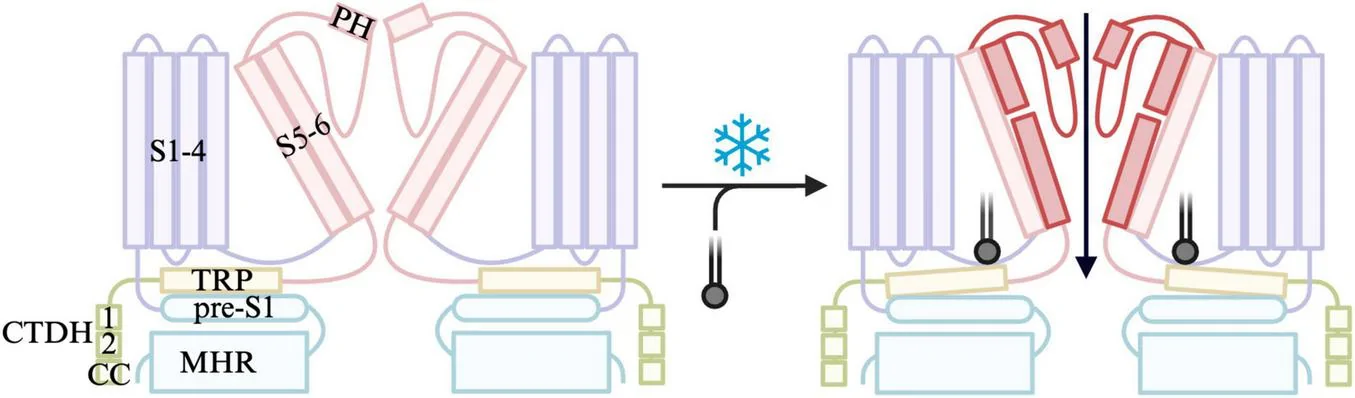
Cold-induced gating of TRPM8. Cold induces PIP_2_ binding and local conformational changes in the outer pore and S6 helices to dilate of the S6 gate, resulting in channel opening. Domains involved in the conformational changes are highlighted in darker colors.

This study captured two distinct architectures of human TRPM8: a fully swapped architecture, in which S6 and the associated PL are domain-swapped with a neighboring subunit, and a semi-swapped architecture, in which S6 and the associated PL remain associated with their cognate S1-S4 bundle. In the semi-swapped architecture, S6 and the PL of one subunit interact with S5 and the PH of the adjacent subunit to form the outer pore intersubunit interface. In the closed state, V915 within the SF is buried in an amphipathic pocket at this interface. Cooling destabilizes V915, exposing surrounding hydrophobic residues and thereby increasing the heat capacity of the region (Table 1; Flowers et al., 2019; Sengupta and Garrity, 2013). HDX-MS provided support for this model by showing that the PH exhibits a decrease in standard folding enthalpy upon cooling, consistent with stabilization of the outer pore intersubunit interface following exposure of hydrophobic residues. Concurrently, S6 shifts upward along its helical axis by approximately one helical turn, leading to reorganization and opening of the lower gate. Cooling also promotes PIP_2_ binding (Figure 4), which stabilizes both the exposed hydrophobic residues at the outer pore intersubunit interface and the upward-shifted S6 conformation, collectively reinforcing the open state. However, the same study reported that channels adopting the fully swapped architecture can also undergo cold-induced opening, even though V915 occupies a different structural environment in that configuration, suggesting that additional mechanisms contribute to cold sensing in TRPM8 (Choi et al., 2026).

**TABLE 1:** Thermodynamic parameters.

Parameter	Parameter definition
enthalpy (*H*)	The sum of a system’s internal energy and the mathematical product of its pressure and volume. *H* = *U* + *PV* *U*: a system’s internal energy; *P*: pressure; *V*: volume.
entropy (*S*)	The ratio of the reversible heat (*q*_rev_) and the kelvin temperature. It is a state function that is a measure of the energy dispersal within a system, determined by the number of system microstates; often described as a measure of the disorder of the system. $\Delta \,S=\frac{q_{r\,e\,v}}{T}$ *q*_*rev*_: reversible heat; *T*: the kelvin temperature.
free energy (*G*)	A thermodynamic property defined in terms of system enthalpy and entropy; all spontaneous processes involve a decrease in *G.* *G* = *H*−*TS* *H*: enthalpy; *S*: entropy; *T*: the kelvin temperature.
heat capacity (*C*)	The quantity of heat (*q*) a body of matter absorbs or releases when it experiences a temperature change (Δ*T*) of 1 degree Celsius (or 1 kelvin). $C=\frac{q}{\Delta \,T}$ *q*: heat absorbed or released by the system; Δ*T*: change in temperature. At constant pressure, the enthalpy change of a system is exactly equal to the heat transferred. So, at constant pressure: $C=\frac{\Delta \,H}{\Delta \,T}\,C\,P\,S\,T\,A\,B\,L\,E\,E\,N\,T\,E\,R\,\Delta$*H*: change in enthalpy; Δ*T*: change in temperature.
*Q*_10_	The ratio of a property measured at two temperatures 10°C apart. $Q_{10}={\left(\frac{R\,2}{R\,1}\right)}^{\frac{10}{T\,2-T\,1}}$ *R*: property measured (such as current); *T*: the kelvin temperature.

Lee et al. resolved the structure of mouse TRPM8 to investigate the gating mechanism of the fully swapped architecture. To capture the open state, the channel was studied in the presence of PIP_2_, menthol and AITC (Lee et al., 2025). As capsaicin allosterically modulates the TRPV1 thermosensitivity, menthol also allosterically modulates the TRPM8 thermosensitivity and increase its temperature activation threshold (Voets et al., 2004). Menthol binds within the S1-S4 bundle and induces rearrangement of an aromatic cluster formed by hydrophobic residues located on CTDH1 and S1. The TRP helix repositions to interact directly with menthol, resulting in an approximately 20° upward swing of the TRP helix and the formation of a 3_10_ helix in S4b. These conformational changes strengthen the coupling between S4 and the TRP helix. This coupling is further reinforced by the interaction of PIP_2_ with S4b, the TRP helix, and MHR4. The movement of S4b disrupts an interaction network involving S4b, the S4-S5 linker, and the neighboring S5, while promoting formation of a new intersubunit interaction network among the S4-S5 linker, the TRP helix, and the neighboring S6 (Lee et al., 2025).

Upon cooling, structural changes occur within the PD, whereas the S1-S4 bundle remains largely unchanged. The menthol binding-induced interaction network involving the S4-S5 linker, the TRP helix, and the neighboring S6 weakens, resulting in rotation of S6, formation of a π-bulge within S6, and extension of S6 by approximately one helical turn. The π-bulge subsequently migrates upward along the helix, accompanied by reorganization of the TRP-S6 linker, an approximately 15° tilt of S6, and further extension of S6 by one additional helical turn. The conformational changes of S6 drive the PH inward and downward, increasing the electronegativity of the pore (Figure 4). Additionally, two hydrogen bonds are formed between S5 and S6, further stabilizing the open conformation (Lee et al., 2025).

Lee et al. captured only the fully swapped architecture and did not observe the semi-swapped configuration reported by Choi et al. Several experimental differences may contribute to this discrepancy. Choi et al. studied human TRPM8, whereas Lee et al. examined mouse TRPM8. Although both used tagged proteins, the tagging strategies differed: Choi et al. fused mEGFP to the N terminus of human TRPM8, whereas Lee et al. attached a Flag-10xHis tag to the C terminus of mouse TRPM8. Lee et al. also introduced an I846V mutation to enhance menthol sensitivity. In addition, Lee et al. were unable to capture an open state induced by cooling alone; instead, the open structure was obtained in the presence of menthol and AITC. Menthol sensitizes the TRPM8 channel, while AITC mitigates its desensitization. Thus, it is unclear whether cooling alone would produce the same intermediate and final open-state structures of the fully swapped architecture (Lee et al., 2025; Choi et al., 2026).

## Discussion

These six studies revealed the temperature-induced open-state structures of two wild-type TRP channels (TRPV3 and TRPM8) (Choi et al., 2026; Nadezhdin et al., 2021), two sensitized TRPV3 mutants (Singh et al., 2019; Mugo et al., 2025), and two channels in the presence of chemical agonists, TRPV1 bound to capsaicin and TRPM8 bound to menthol (Lee et al., 2025; Kwon et al., 2021). Although the sensitized mutants and agonist-bound channels provide valuable tools for capturing intermediate conformational states and investigating temperature-gating mechanisms, they may substantially alter the activation process and therefore not fully recapitulate gating driven solely by temperature. Nevertheless, these findings together provide the first structural insights into the mechanisms by which thermoTRPs are activated by temperature changes.

To open the gate, the PD must undergo conformational rearrangements. All six studies describe structural changes in this region during channel opening. In TRPV3, S6 in the closed structure adopts either an α-helix or a π-helix conformation, depending on the membrane environment (Singh et al., 2019; Nadezhdin et al., 2021; Mugo et al., 2025). When an α-helix is adopted in the closed state, an α-to-π transition occurs during activation in the middle of each S6 helix, leading to rotation of S6b, elongation of S6, and channel opening (Singh et al., 2019). Alternatively, the S4-S5 linker, S5, S6, and the N-terminal portion of the TRP helix may undergo partial unfolding, removing the channel constriction (Mugo et al., 2025). When a π-helix is adopted in the closed state, channel opening involves an upward translation of the S4-S5 linker. S5, S6, and the N-terminal portion of the TRP helix follow the movement of the S4-S5 linker, resulting in an outward displacement of the SF pore-forming regions and increased channel conductance (Figure 2; Nadezhdin et al., 2021). During TRPV1 activation, the outer pore of each subunit tilts and rotates around the membrane, altering subunit interfaces and dilating the SF constriction. In addition, the π-helix-like turn in S6 undergoes rearrangement, resulting in rotation of the S6b segment and channel opening (Figure 3; Kwon et al., 2021). TRPM8 adopts two swapped architectures: semi-swapped and fully swapped structures (Choi et al., 2026). In the semi-swapped structures, S6 remains predominantly an α-helix. During activation, the S6 helix undergoes an upward translation and reorientation, leading to channel opening (Choi et al., 2026). In the fully swapped structures, S6 can adopt either a π-helix or an α-helix conformation in the closed state, depending on the membrane environment. Upon activation, S6 undergoes a series of structural rearrangements to stabilize the open structure, including formation of a π-bulge, upward migration of the π-helix along S6, S6 extension, and S6 rotation. In addition, the PH moves inward and downward, increasing the electronegativity of the pore region (Figure 4; Lee et al., 2025). However, whether the PD rearrangements are themselves temperature dependent remains controversial. Choi et al. and Kwon et al. reported substantial heat-capacity changes associated with this region (Choi et al., 2026; Kwon et al., 2021), suggesting that its conformational transitions are temperature dependent. In contrast, TRPV3 studies, based on closed and open structures of sensitized mutant channels and the requirement of heat for both the closed-to-sensitized and closed-to-open transitions, concluded that PD conformational transitions are only weakly temperature dependent (Singh et al., 2019; Nadezhdin et al., 2021). Although PD rearrangements are indispensable for channel opening, and numerous functional analyses of mutants in this region have shown impaired temperature sensitivity (Yao et al., 2011; Grandl et al., 2010; Grandl et al., 2008; Zhang F. et al., 2018; Kim et al., 2013; Yang et al., 2010; Susankova et al., 2007), these studies are not sufficient to determine whether this region is intrinsically temperature sensitive. Therefore, whether the PD acts as a primary temperature-sensing element remains controversial and requires further investigation.

During activation, the TRP helix also undergoes conformational rearrangements. Multiple studies have reported upward movement of the TRP helix during thermoTRP activation. Singh et al. and Nadezhdin et al. found that in TRPV3, the N-terminal portion of the TRP helix shifts upward during channel opening (Figure 2; Singh et al., 2019; Nadezhdin et al., 2021). Mugo et al. described a similar observation. In the closed state, the TRP helix forms an interaction network with the CD, S2-S3 linker, and ARD. During activation, these interactions are weakened, increasing the flexibility of the N-terminal portion of the TRP helix and promoting partial unwinding at the S6 junction together with slight elongation of the TRP helix N-terminus. These changes shift the S6-TRP junction upward toward the lower gate, thereby dilating the gate (Figure 2; Mugo et al., 2025). In TRPM8, an upward rotation of the TRP helix has also been observed (Figure 4; Lee et al., 2025). Additional TRP helix rearrangements have also been reported. For example, the TRP helix of TRPV3 shortens by approximately two helical turns during activation (Singh et al., 2019). The TRP helix moves closer to the pore during TRPV1 activation (Kwon et al., 2021). However, it is unclear whether these conformational rearrangements of the TRP helix are directly driven by temperature or instead represent downstream structural changes that propagate temperature-induced conformational transitions.

Based on studies of TRPV1 and TRPV3, temperature induces global structural rearrangements characterized by overall channel contraction (Singh et al., 2019; Nadezhdin et al., 2021; Mugo et al., 2025; Kwon et al., 2021). During TRPV3 activation, vertical compression results from rotation of the intracellular domains toward the TMD (Nadezhdin et al., 2021; Mugo et al., 2025). Lateral contraction toward the pore axis is driven by inward displacement of the TMD toward the central pore, together with counter-rotation of the TMD relative to the intracellular domains (Nadezhdin et al., 2021; Mugo et al., 2025). During TRPV1 activation, the ARD, the CD, the S1-S4 domain, and the C-terminal portion of the S4-S5 linker rotate toward the channel center. Concurrently, the TRP helix moves closer to the pore, and the CD and ARD move closer to the TRP helix (Kwon et al., 2021). During this process, most domains, including the ARDs and the S1-S4 bundle, do not undergo substantial local conformational changes but instead move as rigid bodies. Since TRPM8 studies have not reported whether a comparable global structural rearrangement occurs (Lee et al., 2025; Choi et al., 2026), it is unclear whether such temperature-induced rigid-body rearrangements and global structural changes represent a conserved mechanism underlying thermoTRP activation.

Lipid binding or dissociation may contribute to temperature-induced activation (Rohacs, 2024). In TRPM8, cooling may enhance lipid binding. For example, PIP_2_ binding stabilizes S6 at a position shifted upward by approximately one helical turn and stabilizes the open conformation (Figure 4; Choi et al., 2026). In contrast, heating may involve lipid dissociation in some thermoTRPs. In TRPV3, two lipid densities are observed in the closed state. These lipid densities reduce in the sensitized state and are absent in the open state, suggesting that lipid dissociation accompanies TRPV3 activation (Figure 2, upper panel; Nadezhdin et al., 2021; Singh et al., 2019). In TRPV1, heating induces rearrangement of lipids at the subunit interface, which facilitates outer pore rearrangements (Kwon et al., 2021). Lipid binding or dissociation may modulate interdomain and/or intersubunit interactions, triggering global structural rearrangements. For example, in TRPM8, PIP_2_ interacts with S4b, the TRP helix, and MHR4. Together with menthol binding, these interactions alter the helicity of the S4-S5 linker, rotate the TRP helix, and rearrange S6 (Lee et al., 2025). Reciprocally, interdomain and/or intersubunit rearrangements may promote lipid binding or dissociation, suggesting a potential bidirectional relationship between lipid occupancy and channel conformation. Of importance, lipid binding or dissociation alone is not sufficient to open thermoTRPs, even though this process itself may be temperature dependent (Lee et al., 2025; Nadezhdin et al., 2021). Moreover, Mugo et al. did not observe lipid rearrangement during heat activation of TRPV3 (Figure 2, lower panel), suggesting that lipid association may vary under different conditions (Mugo et al., 2025).

There are two competing models for the molecular mechanism underlying temperature sensing in TRP channels: the localized model and the distributed model (Feng, 2014). The localized model refers to the existence of a discreate domain, a temperature sensor, that is responsible for temperature sensing. The distributed model proposes that temperature sensing is distributed across the entire protein, leading to global conformational changes. Six studies discussed in this review indicate that temperature changes induce conformational rearrangements involving multiple domains and often distributing to the entire protein (Choi et al., 2026; Kwon et al., 2021; Lee et al., 2025; Mugo et al., 2025; Nadezhdin et al., 2021; Singh et al., 2019), supporting the distributed model. However, intermediate structural snapshots show that these global changes do not occur simultaneously but instead represent a propagating conformational wave that originates from a specific region and subsequently spreads across the entire protein. Therefore, the initiation site of this conformational propagation may represent a functional temperature-sensing element. However, this element differs fundamentally from the classical localized sensor. In the localized model, a sensor generally refers to a discrete structural domain that directly detects a stimulus and undergoes a conformational change to regulate channel gating, such as the voltage sensor in voltage-gated ion channels. To distinguish this mechanism from the classical localized sensor model, Singh et al. and Mugo et al. proposed the term “latch.” Here, we use this term to describe multiple structural elements that coordinate to initiate temperature-induced conformational rearrangements in thermoTRPs. Temperature-sensing latches exhibit two distinct properties from the classic localized sensors. (1) A latch may encompass multiple domains. For example, in TRPV3, it may involve regions that undergo secondary structural changes upon heating, including the S2-S3 linker, N-terminus, and C-terminus. It may also include the lipid-binding sites (Nadezhdin et al., 2021). (2) A latch may represent an interaction network, such as the intersubunit interface or the interface between the TMD and intracellular domains (Choi et al., 2026; Kwon et al., 2021; Lee et al., 2025; Mugo et al., 2025; Nadezhdin et al., 2021; Singh et al., 2019). Of importance, these two properties are closely interconnected. For instance, in TRPV3, conformational changes in the N- and C-termini are accompanied by remodeling of the interaction network at the intersubunit interface.

Cryo-EM analyses of temperature-induced activation of TRP channels provide direct structural evidence for how thermoTRPs are activated in response to temperature changes. The six studies discussed in this review represent an important first step toward understanding this process. However, they capture only a limited number of discrete snapshots along a complex and dynamic conformational trajectory. Future studies will require higher temporal resolution to resolve the complete conformational wave underlying temperature gating. Beyond TRP channels, cryo-EM studies of other temperature-responsive molecules are far behind, such as the temperature-activated ionotropic receptor complexes and gustatory receptor paralogs identified in *Drosophila* (Ni et al., 2013; Ni et al., 2016; Knecht et al., 2016; Hernandez-Nunez et al., 2021). Extending structural analyses to these molecules and resolving their temperature-driven conformational changes will be essential for establishing a more general and unified framework for understanding how temperature activates thermoresponsive molecules.

## Supplementary material

Supplementary Table 1 Comparison of the six cryo-EM studies capturing temperature-induced open conformations of thermoTRP channels. The table summarizes the thermoTRP channels examined, expression and protein purification strategies, cryo-EM membrane environments, chemical and lipid association conditions, temperature treatment used to obtain open structures, structural states captured (closed, intermediate, and open), key conformational changes, and the limitations of each study.
